# Combining Motion Analysis and Microfluidics – A Novel Approach for Detecting Whole-Animal Responses to Test Substances

**DOI:** 10.1371/journal.pone.0113235

**Published:** 2014-12-02

**Authors:** Tabitha S. Rudin-Bitterli, Oliver Tills, John I. Spicer, Phil F. Culverhouse, Eric M. Wielhouwer, Michael K. Richardson, Simon D. Rundle

**Affiliations:** 1 Marine Biology and Ecology Research Centre, School of Marine Science and Engineering, University of Plymouth, Drake Circus, Plymouth, United Kingdom; 2 Centre for Robotics and Neural Systems, School of Computing and Mathematics, University of Plymouth, Drake Circus, Plymouth, United Kingdom; 3 Sylvius Laboratory, Institute of Biology, Leiden University, Leiden, The Netherlands; 4 Syntecnos, Leiden, The Netherlands; Oregon State University, United States of America

## Abstract

Small, early life stages, such as zebrafish embryos are increasingly used to assess the biological effects of chemical compounds *in vivo*. However, behavioural screens of such organisms are challenging in terms of both data collection (culture techniques, drug delivery and imaging) and data evaluation (very large data sets), restricting the use of high throughput systems compared to *in vitro* assays. Here, we combine the use of a microfluidic flow-through culture system, or BioWell plate, with a novel motion analysis technique, (sparse optic flow - SOF) followed by spectral analysis (discrete Fourier transformation - DFT), as a first step towards automating data extraction and analysis for such screenings. Replicate zebrafish embryos housed in a BioWell plate within a custom-built imaging system were subject to a chemical exposure (1.5% ethanol). Embryo movement was videoed before (30 min), during (60 min) and after (60 min) exposure and SOF was then used to extract data on movement (angles of rotation and angular changes to the centre of mass of embryos). DFT was subsequently used to quantify the movement patterns exhibited during these periods and Multidimensional Scaling and ANOSIM were used to test for differences. Motion analysis revealed that zebrafish had significantly altered movements during both the second half of the alcohol exposure period and also the second half of the recovery period compared to their pre-treatment movements. Manual quantification of tail flicking revealed the same differences between exposure-periods as detected using the automated approach. However, the automated approach also incorporates other movements visible in the organism such as blood flow and heart beat, and has greater power to discern environmentally-driven changes in the behaviour and physiology of organisms. We suggest that combining these technologies could provide a highly efficient, high throughput assay, for assessing whole embryo responses to various drugs and chemicals.

## Introduction

Evaluation of embryonic and larval behaviour is an important tool for toxicological, pharmacological and biomedical studies as it enables the non-invasive investigation of sub-lethal effects on the embryonic phenotype [1]–[3]. However, behavioural screening assays bring major challenges in terms of data collection and data evaluation, both of which are highly time-consuming activities that could prohibit the use of high throughput systems compared to *in vitro* assays. These limitations are being addressed by technological innovations in imaging, culture techniques and drug delivery. Furthermore, the bioinformatics resources available to analyze behavioural data are rapidly expanding [4].

Zebrafish (*Danio rerio*) embryos have recently been proposed as an *in vivo* model to bridge the gap between simple *in vitro* assays and biological validation in whole animals such as rodents [5]–[7]. Their small size, transparency during the embryonic period and rapid, external development make zebrafish particularly suitable for high throughput behavioural assays [8], [9]. Zebrafish embryos have recently been cultured successfully in a microfluidic flow-through system constructed from borosilicate glass (BioWell plate) [6]. This BioWell plate has several advantages over the standard (e.g. 96-wells) microtiter plates commonly used in zebrafish screening experiments [6]. Most notably it enables rapid exposure of embryos to numerous treatments using only small volumes of treatment solution without causing major disruption to the environment of the embryo. Furthermore, a rapid change of treatment is possible, by flushing the embryos with control buffer and investigating the recovery responses. The BioWell plate also concentrates embryos into a smaller physical area than is possible with 96-well microtitre plates. This makes it easier to image large numbers of individuals, because it reduces the seek time and means that more pixels coincide with the embryo itself rather than with dead space.

Although the BioWell plate provides the opportunity to develop a system for the automated collection of behavioural data from toxicological and biomedical assays, the manual evaluation of such data is time-consuming, creating a bottleneck that can limit the throughput of embryos in an experiment. Furthermore, manual observations are prone to human error [10]. Many behavioural endpoints are difficult to define, making the consistent reproducibility and comparison of results between different research groups problematic [11].

Developments in computer-assisted image analysis have the potential to overcome these limitations. Some computer-assisted motion analysis software systems are already commercially available and have been used successfully to quantify the behaviour of adult zebrafish [12], sperm [13]–[15], invertebrate larvae [16], algal spores [17], [18] and bacteria. However, their use with embryos has been limited, as they rely on gross changes in movement patterns and are, potentially, not suitable for quantifying and distinguishing the more subtle movements typical of embryos [16].

Recently, a novel motion analysis technique was used to quantify motion patterns in aquatic embryos that incorporate both gross movements (e.g. tail flicking in fish and spinning in molluscan embryos) and other more subtle parameters such as heart beat, blood flow and muscle flexing [19]. This technique is based on frame-to-frame motion analysis [20], which generates data that are analyzed using a spectral frequency analysis to quantify the extent to which embryos move at different frequencies. This approach does not rely on the gross movement of an organism, but instead identifies and tracks multiple points in the embryo from one frame to the next. It provides an integrative measure of the movement of an embryo, incorporating all types of motion into a single analysis.

Here, we report the first combined use of the BioWell plate with this integrative spectral motion analysis technique. We used zebrafish embryos as our model given their increasing importance in drug screening and their extensive use in behavioural bioassays; ethanol was used to produce a set of distinct behavior patterns that differed from those present during normal development. Although ethanol was chosen because of its biomedical importance [21]–[24] the aim of this current study was not to assess the effect of ethanol per se. Instead we assessed whether combining these technologies could facilitate the detection of differences in behaviour in zebrafish embryos before, during and after a short-term exposure to alcohol, and how this compared with detection using manual observation.

## Methods

### Embryo preparation

All animal experimental procedures were conducted in accordance with local and international regulations and Plymouth University's Research Ethics Policy. Zebrafish embryos used in this study were no more than 5 days post fertilisation and therefore in accordance with the Animal (Scientific Procedure) Act 1986 no license was required for this experiment.

Zebrafish (*Danio rerio*) eggs from a wild type (WIK) population were obtained from the zebrafish facility at Plymouth University by overnight, random pair-wise mating. The next morning, they were transferred to glass beakers (vol.  = 200 ml) each containing aerated, deionized water (T = 27°C) [23]. The beakers were placed into a water bath (Model DMU26, Fisherbrand, Leicestershire, UK) maintained at T = 27°C. Periodically (approx. every 30 min), the water in the beakers was changed and any dead embryos removed. Three hours before the experiment (18.5 h post fertilization), the beakers containing the embryos were removed from the water bath and moved to a controlled temperature environment (T = 20°C±0.5°C), where the exposures described below would take place, and left to acclimatize for 3 h.

### Experimental set-up

Before the experiment commenced, a syringe pump (Model NE-300, New Era Pump Systems Inc., Farmingdale, USA) containing aerated, deionized water [23] was attached to the inflow port of the microfluidic flow chip (“BioWell plate”, 32 round wells [diam.  = 2 mm; vol. = 10 µl]; Syntecnos: Leiden, the Netherlands) by means of phenyl/methyl deactivated capillary tubing (150 µm inner diameter and 375 µm outer diameter; BGB Analytik AG: Schlossboeckelheim, Germany) and cross interconnectors. The syringe pump circulated the water through the BioWell plate (flow rate: 4 µl well^−1^ min^−1^), filling all arrays of wells simultaneously (connected in parallel by channels) and the water was ejected through the outflow port after circulation through the wells. The water was not recirculated. Water circulation was then temporarily halted and fifteen *Danio rerio* embryos at the 22-somite stage [25] with intact chorions were individually loaded into separate wells of the BioWell plate. The lid was sealed (as described by Wielhouwer et al. [6]) and water flow was restarted.

**Figure 1:**
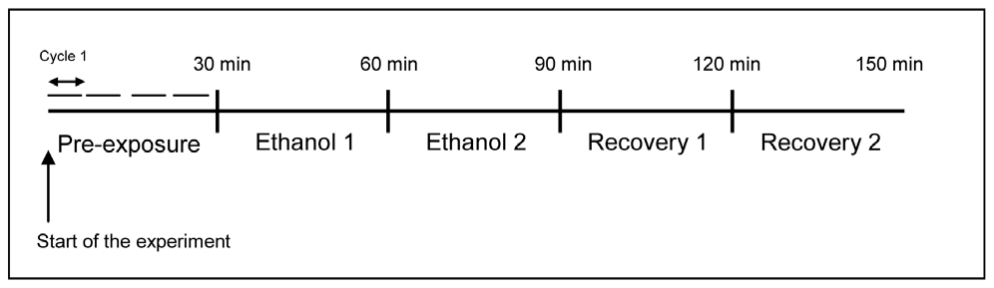
Schematic representation of the experimental design. One cycle represents the time period taken to record all 15 embryos once (7.5 min). The experiment is divided into five time periods: ”pre-exposure period” (recording cycle 1–4: aerated, deionized water), “ethanol period” (divided into two groups “ethanol 1” (recording cycle 5–8) and “ethanol 2” (recording cycle 9–12); 1.5% ethanol solution) and “recovery period” (divided into two groups “recovery 1” (recording cycle 13–16) and “recovery 2” (recording cycle 17–20); aerated, deionized water).

The BioWell plate was imaged using a custom imaging system (described below) and embryos were acclimatized for 60 min to both water flow and lighting of the imaging system before beginning the experiment. Embryos within the BioWell plate were exposed to the following regime: 30 min exposure to aerated, deionized water (pre-exposure period); then 60 min exposure to a 257 mmol.l^−1^ (1.5% v/v) ethanol solution in aerated, deionized water (ethanol period) and finally 60 min exposure to aerated, deionized water (recovery period; Fig. 1). The ethanol and recovery periods were later divided into two sub-periods, each 30 min long, to facilitate statistical analysis (“ethanol 1” and “ethanol 2” and “recovery 1” and “recovery 2” respectively; see Analytical approach and Fig. 1 for further information).

### Imaging setup

Image sequences of zebrafish in the pre-exposure, ethanol and recovery periods were captured using a four megapixel shutterless camera (Pike 421B, Allied Vision Technology, Stadtroda, Germany) operating at 1024×800 pixels and 15 Hz. The camera was connected to a zooming lens system (VHZ100R, Keyence, Milton Keynes, United Kingdom) operating at x100 magnification. The camera and lens were inverted beneath an Optiscan XY motorized stage system (Prior Scientific, Cambridge, United Kingdom) which was controlled and synchronized with the camera using the Image J plugin, Micromanager 1.3 [26].

Embryos were imaged individually for 30 sec and this recording cycle was repeated every 7.5 minutes for the duration of the experiment. During the experiment (2.5 h), four recording cycles were completed during the pre-exposure period and eight cycles were recorded during each of the ethanol and recovery periods (Fig. 1).

### Motion analysis

The image sequences were analyzed off-line for Sparse Optical Flow (SOF) using the OpenCV toolkit [27]. Optic flow is the distribution of apparent velocities of movement of brightness patterns in a picture. Corner features are extracted from each picture and optic flow then tracks each feature point from one frame to the next by iterative approximation. A velocity variance is assigned to each displacement of features between two consecutive frames. The method is not specific to the embryo and can occasionally be complicated by the movement of other features, such as the chorion. Here, four measures of motion were extracted for each frame-to-frame comparison: i) positive (clockwise) and ii) negative (anti-clockwise) angles of rotation; and iii) rho and iv) theta angular changes (in polar coordinates) to the centre of mass. Sparse optic flow measurements were saved as a comma separated value (CSV) file [19].

Data were analyzed for spectral content using the Discrete Fourier Transform (DFT) [28], which separates the frame-to-frame motion data into different temporal frequency bins (224 bins in total), using the R language version 2.13.0 [29]. Each image sequence was 30 sec long and frequency analysis can therefore reveal repetitive oscillations from 7.5 Hz to 0.0333 Hz in the frame-to-frame motion output generated by each image sequence. These are calculated for each image sequence. Eighteen specific frequency bins were further analyzed from the DFT: [1] 29.87–14.93 sec; [2] 9.96–7.47 sec; [3] 5.97–4.98 sec; [4] 4.27–2.72 sec; [5] 2.49–1.30 sec; [6] 1.24–1.07 sec; [7] 1.03–0.85 sec; [8] 0.83–0.73 sec; [9] 0.71–0.64 sec; [10] 0.62–0.51 sec; [11] 0.45–0.40 sec; [12] 0.39–0.32 sec; [13] 0.31–0.27 sec [14] 0.27–0.24 sec; [15] 0.23–0.21 sec; [16] 0.21–0.19 sec; [17] 0.19–0.17 sec; [18] 0.16–0.13 sec.

### Analytical approach

The data generated by the DFT comprised 18 frequency bins for each of the SOF parameters (i.e. positive and negative angles of rotation and rho-theta angular changes in polar coordinates to the centre of mass) for each individual (Dataset S1). These data were transformed (Log X+1) and analyzed using multivariate procedures in the PRIMER statistical program [30]. First, a Bray-Curtis similarity matrix was constructed. Then, the motion analysis data were divided into five time periods: i) “pre-exposure” (recording cycle 1–4 - aerated, deionized water); ii) “ethanol 1” (recording cycle 5–8 - 1.5% ethanol solution); iii) “ethanol 2” (recording cycle 9–12 - 1.5% ethanol solution); iv) “recovery 1” (recording cycle 13–16 - aerated, deionized water); and v) “recovery 2” (recording cycle 17–20; aerated, deionized water) (Fig. 1). The degree of similarity between the five groups was tested using ANOSIM (also within the PRIMER package [30]). A Multidimensional Scaling (MDS) plot was generated using PRIMER from the mean values of the 72 parameters of each of the five groups after these were logarithmically transformed and a Bray and Curtis similarity matrix was calculated.

### Manual observations

**Figure 2:**
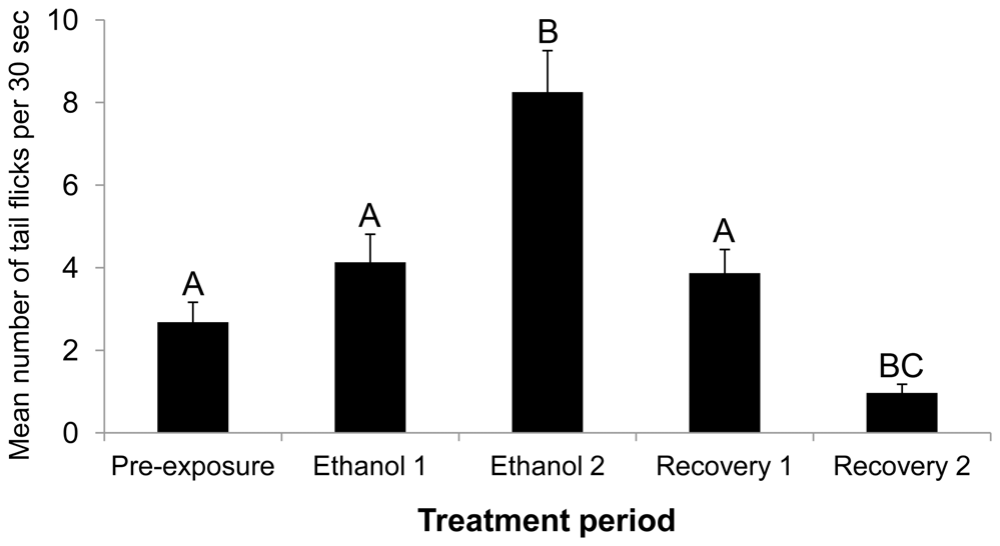
Mean number of tail flicks performed by *Danio rerio* embryos during the five treatment periods as obtained through manual quantification (mean ± 1.S.E.). Values with different letters are significantly different (Repeated Measures ANOVA followed by Bonferroni pair wise comparisons, p<0.05). For significance levels please refer to Table 1.

**Figure 3:**
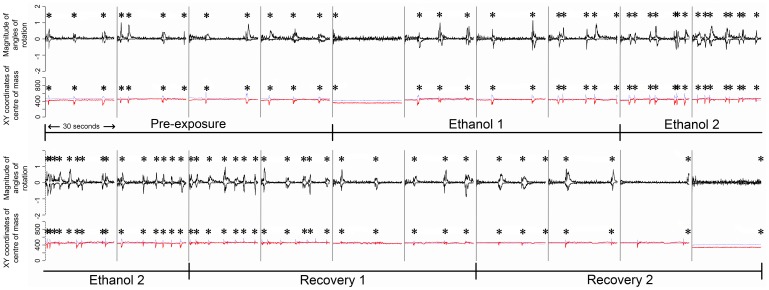
Optic flow movement patterns of an individual zebrafish embryo (individual No. 4) at the 22-somite stage for the time period of the whole experiment (pre-exposure, ethanol and recovery periods). The stars indicate manually observed tail-flick movements.

Image sequences from the treatments were observed manually and measurements were made of tail flicking frequency for individual *D. rerio* embryos observable in each 30 sec video segment (Dataset S2). Differences in tail flick frequency between the five exposure periods were tested for using a repeated-measures ANOVA performed using MINITAB [31]. Bonferroni pair-wise comparisons (also using MINITAB) were used to identify specific differences between the five exposure periods. The level of significance of both the DFT and the manual analysis statistical analyses were compared to assess the efficiency of the motion analysis technique. Furthermore, the time point of each manually observed tail flick was noted (Fig. 2) for every embryo to allow verification of the tail flick movement patterns produced by the optic flow analysis being the cause of the dominant peaks seen in the frame-to-frame motion analysis output (Fig. 3).

## Results

**Figure 4:**
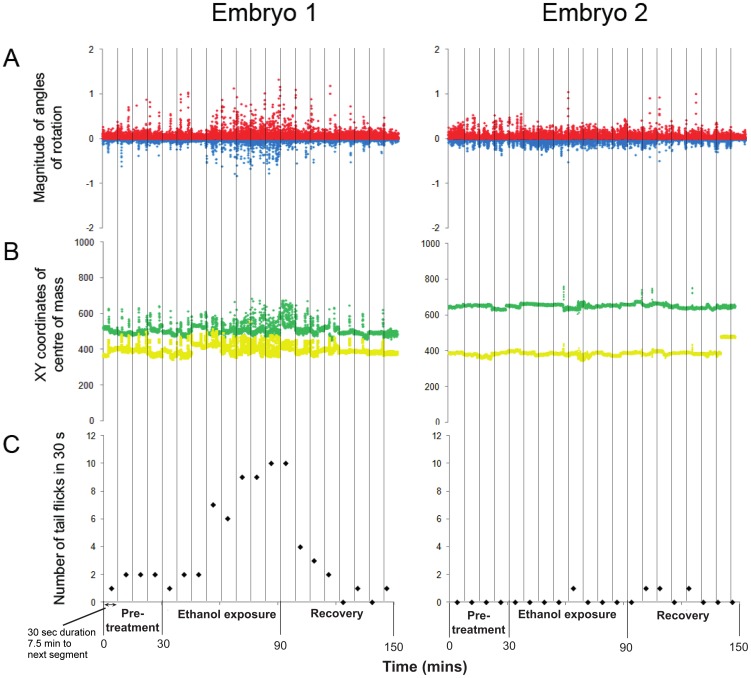
Movement patterns of two embryos during the time period of the experiment. Embryo 1 and Embryo 2 exhibited different movement responses to ethanol exposure, Embryo 1 increasing tail flick frequency markedly whereas the response of Embryo 2 was more subtle. A+B: Frame by frame optic flow parameters (red – positive angle movements, blue – negative angle movements, green – X coordinate of centre of mass, yellow – Y coordinate of centre of mass). C: tail flick frequency (tail flicks per 30 sec).

Embryos performed tail flicking behaviour (2.68±0.48 tail flicks per 30 sec - mean ± S.E) during the pre-exposure period (30 min in aerated, deionized water). This movement was accurately tracked by optic flow (Table 1 and Fig. 3) and could be visualized as peaks in positive and negative movement and the X and Y coordinates of the centre of mass of the embryo in the movement patterns produced (Figs 3 & 4).

**Table 1:** Comparison of significance levels of pair-wise differences in embryos from the five experimental treatments (pre-exposure, ethanol 1 and 2 as well as recovery 1 and 2) obtained from the automated motion analysis technique (ANOSIM of Bray and Curtis similarity matrices calculated from the results of Discrete Fourier Transform on frame-to-frame motion parameters), and the manual quantification (Repeated Measures ANOVA, followed by Bonferroni pair wise comparisons) of tail flick frequency performed by *Danio rerio* embryos.

	Pre-exposure		Ethanol 1		Ethanol 2		Recovery 1	
	Automated	Manual	Automated	Manual	Automated	Manual	Automated	Manual
**Ethanol 1**	ns	ns						
**Ethanol 2**	***	***	***	***				
**Recovery 1**	ns	ns	ns	ns	**	***		
**Recovery 2**	**	*	***	**	***	***	***	*

***−p ≤ 0.001, **−p ≤ 0.01, *−p ≤0.05, ns - not significant.

During the first 30 min of ethanol exposure (ethanol 1), embryos showed a gradual increase in the number of tail flicks per unit time, reaching a maximum (8.25±1.01 tail flicks per 30 sec) after 40–50 min exposure (ethanol 2; Fig. 2). This increase in tail flick frequency could be identified in optic flow as an increase in the number of large peaks in positive and negative movement (Fig. 3). Furthermore, the embryos not only increased their tail flick frequency, but the tail flicks were more vigorous during ethanol 2. This caused the embryos to move within the chorion, and this, in turn, resulted in changes to the calculated X and Y coordinates of the centre of the mass of the embryos (Figs 3 & 4).

Replacing ethanol with deionized water (recovery 1) led to a gradual decrease in tail flick frequency (3.87±0.57 tail flicks per 30 sec). After 30 to 60 min of exposure to deionized water (recovery 2), tail flick frequency was significantly reduced (0.97±0.21 tail flicks per 30 sec) (Table 1) compared to the pre-exposure period, suggesting that there might be persistent effects of ethanol exposure on the embryos. An alternative explanation is that the ethanol remained within the chorion in the perivitelline fluid, and therefore continued to exert an effect on the embryo (see the Ali *et al*. (2011) [19] paper for NMR metabolomics profiling of embryos exposed to 10% ethanol).

**Figure 5:**
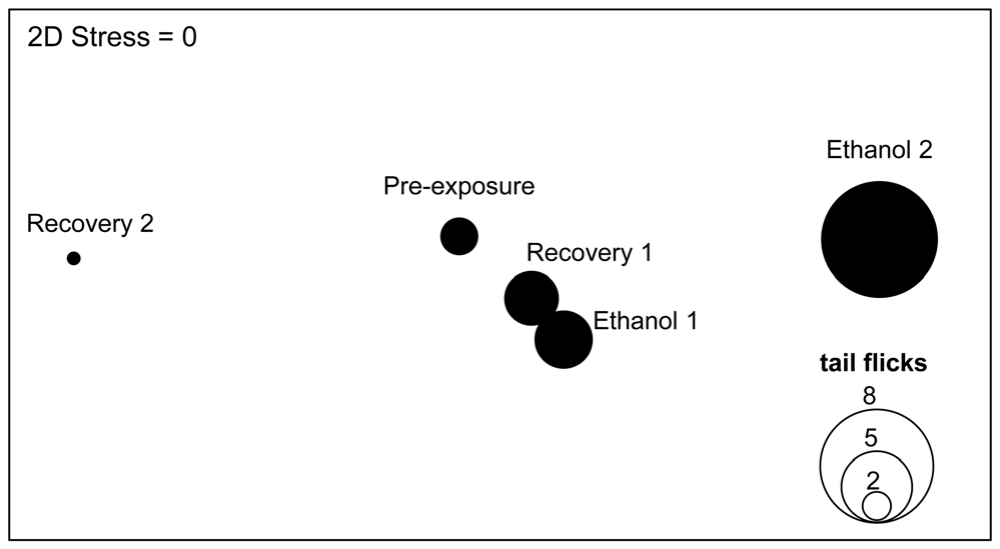
Multidimensional scaling plot in two dimensions using Bray and Curtis similarities, performed on logarithmically transformed data consisting of 18 frequency bins for each of (i) negative angle, (ii) positive angle, (iii) centre of mass – rho, and (iv) centre of mass – theta. The mean values of each of the five groups (pre-exposure, ethanol 1 and 2, recovery 1 and 2) were used. Dissimilarities in the motion of embryos within the five experimental periods is signified by the distance between them. The bubble size reflects the average frequency of tail flicks of the 15 embryos for each group.

Comparison of the DFT with the manual analysis showed that the two analyses matched very closely. In instances where the manual analysis revealed significant differences in embryonic movements between treatment periods, the DFT also did so (Table 1). Similarly, when the manual analysis did not reveal significant differences, neither did the DFT (Table 1). Embryonic movements significantly differed between all of the treatment periods except between the ethanol 1 period and pre-exposure and between the recovery 1 and the pre-exposure periods (Fig. 5, Table 1). During the course of the experiment no morphological abnormalities were observed, although it should be noted that no observations were made beyond the experimental period.

## Discussion

The combination of a microfluidic flow-through system (BioWell plate) with a motion analysis technique that incorporates all visible embryonic movements was reliably able to identify behavioural changes of zebrafish embryos exposed to ethanol. A common limitation in many biological studies is the considerable time required to analyze the data generated by an experiment. Ironically, this limitation can be exacerbated by technological advances facilitating the acquisition and accumulation of increasingly larger and more detailed biological datasets. The motion analysis technique we use here offers one solution to this issue by providing an innovative automated approach that quantifies visible movements of embryos, providing a reliable and biologically-relevant indicator of the response of an embryo to a particular treatment [19]. This study demonstrated that the combined use of the Biowell plate with motion analysis provides the capability and sensitivity to detect even subtle changes in embryonic behaviour and physiology.

Comparison of manual quantification of movements with the motion analysis output generated from the same video suggests that this motion analysis technique provides an accurate description of embryonic movements. Tail flicks were the largest and most distinctive movement observed in the zebrafish and were visible in the frame-to-frame motion analysis output as the largest peaks visible during the experiment. Quantifying tail flick frequency was possible by simply counting the number of large peaks in positive and negative frame-to-frame motion output and these corresponded precisely to the occurrence of tail flicks recorded by manual observation (Fig. 3). Our results suggest that an acute exposure to 1.5% ethanol stimulated a significant increase in the rate of tail flicking. This finding is supported by other studies investigating behavioral effects of ethanol in zebrafish [21], [22], [32]–[35]. Acute exposure of zebrafish to ethanol at low concentration (1–2%) has been found to stimulate locomotion, whereas exposure at higher concentrations (4%) decreased locomotor activity [35]. The most widely used system for quantifying the behavior of zebrafish is probably the EthoVision system [36], which encompasses both the hardware and software for tracking the gross movements of zebrafish. EthoVision does also now include the facility to specify the location of the heart in the image and changes in the pixels at this location are then used to extract heart rate. The technique we present here differs from EthoVision by not relying on gross movements of the zebrafish, but instead tracking and quantifying all movements in both internal and external features that are visible in the images recorded. A limitation of the current system compared to Ethovision is that, high resolution video is required in order to accurately track the internal features of the embryos, which means that simultaneous imaging of all wells of the Biowell plate is not optimal. This limitation is offset by the ability to study subtle embryonic movements and thereby provide a holistic quantification of the movement patterns exhibited by an embryo. The technique used here could be applicable for use in high throughput screening for applications such as drug discovery and toxicology. The use of automated analyses facilitates quantifying the response of large numbers of embryos and reduces potential data bottlenecks. Even so, large quantities of data can be generated quickly with such approaches and progress is being made in the field of pharmacology in developing tools to effectively store large behavioral datasets and to mine these (reviewed in [37])

The approach we use here is novel in that it incorporates two technological advances: the BioWell plate, a microfluidic flow-through culture system for aquatic organisms, and integrative spectral frequency analysis. The BioWell plate provides an effective method for exposing zebrafish embryos to water soluble compounds and has the advantage over the typically used microtitre plates that both pre-treatment and recovery can be monitored, with minimal disturbance to the embryo. In addition, the BioWell plate setup has no air compartment at the top of the well and therefore prevents condensation typically observed on the top lid of classical microtiter well plate setups. This has the advantage of both excluding false negative results caused by cross contamination of treatments between wells and improved quality of imaging due to light not being scattered by condensate. The motion analysis technique we use does not require specific movement patterns and therefore such analysis could be extended to embryos belonging to other phyla in order to evaluate changes to movement in response to various treatments such as environmental stimuli, drugs and other chemicals. Tills *et al.* [19] demonstrated that the motion analysis technique used here was effective at identifying stress responses in various developmental stages of zebrafish, the African clawed frog *Xenopus laevis* and the freshwater pond snail *Radix balthica*. This in turn suggests that the combined use of this motion analysis technique with the BioWell plate may provide a powerful tool for high throughput screening of whole-embryo responses of various aquatic organisms to potential stressors.

## Conclusions

Whilst behavioural screening of embryos is an essential tool for assessing the effects of test substances *in vivo*, the scale of such experiments is often restricted due to challenges in both data collection and evaluation. Here we present an approach that successfully addresses both of these limitations. We show that the combined use of a microfluidic flow-through culture system with an integrative spectral frequency analysis was reliably able to identify behavioural changes of zebrafish embryos exposed to ethanol. The results of manual quantification of movement and motion analysis data matched very closely, demonstrating that this technique can successfully detect even subtle whole-animal responses. Our results suggest that the combination of the two techniques could facilitate the high throughput screening of embryos *in vivo*.

## Supporting Information

Dataset S1 **Results of Discrete Fourier Transformation performed on each video recorded for the parameters Negative angle of rotation, positive angle of rotation, rho angular change to the centre of mass and theta angular change to the centre of mass.** For further information please refer to the Methods.
(XLSX)

Dataset S2 **Manual counts of the numbers of tail flicks performed during each video sequence.**
(XLSX)

## References

[1] Brittijn SA, Duivesteijn SJ, Belmamoune M, Bertens LFM, Bitter W, et al (2009) Zebrafish development and regeneration: new tools for biomedical research. Int J Dev Bio 53:835–850. 19557689 10.1387/ijdb.082615sb

[2] Champagne DL, Hoefnagels CCM, de Kloet RE, Richardson MK (2010) Translating rodent behavioral repertoire to zebrafish (*Danio rerio*): Relevance for stress research. Behav Brain Res 214:332–342. 20540966 10.1016/j.bbr.2010.06.001

[3] Gerlai R (2010) High-throughput behavioral screens: the first step towards finding genes involved in vertebrate brain function using zebrafish. Molecules 15:2609–2622. 20428068 10.3390/molecules15042609 PMC6257226

[4] Peng HC (2008) Bioimage informatics: a new area of engineering biology Bioinformatics. 24:1827–1836. 10.1093/bioinformatics/btn346 PMC2519164 18603566

[5] Lieschke GJ, Currie PD (2007) Animal models of human disease: zebrafish swim into view. Nat Rev Genet 8:353–367. 17440532 10.1038/nrg2091

[6] Wielhouwer EM, Ali S, Al-Afandi A, Blom MT, Riekerink MBO, et al (2011) Zebrafish embryo development in a microfluidic flow-through system. Lab Chip 11:1815–1824. 21491052 10.1039/c0lc00443j

[7] Pardo-Martin C, Chang TY, Koo BK, Gilleland CL, Wasserman SC, et al (2010) High-throughput in vivo vertebrate screening. Nat Methods 7:634–646. 20639868 10.1038/nmeth.1481 PMC2941625

[8] Parng C, Seng WL, Semino C, McGrath P (2002) Zebrafish: A preclinical model for drug screening. . Assay Drug Dev Technol 1:41–48. 15090155 10.1089/154065802761001293

[9] Ali S, Champagne DL, Spaink HP, Richardson MK (2011) Zebrafish embryos and larvae: A new generation of disease models and drug screens. Birth Defects Res C 93:115–133. 10.1002/bdrc.20206 21671352

[10] Gerlai R (2005) 5th International Conference on Methods and Techniques in Behavioral Research, Wageningen, Netherlands.

[11] Hicks C, Sorocco D, Levin M J (2006) Automated analysis of behavior: A computer-controlled system for drug screening and the investigation of learning. Neurobiol 66:977–990. 10.1002/neu.20290 16779826

[12] Williams LR, Wong K, Stewart A, Suciu C, Gaikwad S, et al (2011) Behavioral and physiological effects of RDX on adult zebrafish. Comp. Biochem Phys C 155:33–38. 10.1016/j.cbpc.2011.02.010 21382508

[13] Larsen L, Scheike T, Jensen TK, Bonde JP, Ernst E (2000) Computer-assisted semen analysis parameters as predictors for fertility of men from the general population. Hum Reprod 15:1562–1567. 10875866 10.1093/humrep/15.7.1562

[14] Suzuki T, Shibahara H, Tsunoda H, Hirano Y, Taneichi A (2002) Comparison of the Sperm Quality Analyxer IIC variables with the computer-aided sperm analysis estimates. Int J Androl 25:49–54. 11869377 10.1046/j.1365-2605.2002.00324.x

[15] Liu QH, Li J, Xiao ZZ, Ding FH, Yu DD (2007) Use of computer-assisted sperm analysis (CASA) to evaluate the quality of cryopreserved sperm in red seabream (Pagrus major). Aquaculture 263:20–25.

[16] Amsler MO, Amsler CD, Rittschof D, Becerro MA, McClintock JB (2006) The use of computer-assisted motion analysis for quantitative studies of the behaviour of barnacle (*Balanus amphitrite*) larvae. Mar. Freshw. Behav. Physiol 39:259–268.

[17] Greer SP, Iken K, McClintock JB, Amsler CD (2006) Bioassay-guided fractionation of antifouling compounds using computer-assisted motion analysis of brown algal spore swimming. Biofouling 22:125–132. 16581677 10.1080/08927010600602082

[18] Iken K, Greer SP, Amsler CD, McClintock JB (2003) A new antifouling bioassay monitoring brown algal spore swimming behaviour in the presence of echinoderm extracts. Biofouling 19:327–334. 14650087 10.1080/08927010310001612045

[19] Tills O, Bitterli TS, Culverhouse P, Spicer JI, Rundle SD. (2013) A novel application of motion analysis for detecting stress responses in embryos at different stages of development. BMC Bioinformatics 14:37. 23374982 10.1186/1471-2105-14-37 PMC3573997

[20] Senst T, Eiselein V, Sikora T (2010) II-LK – A real time implementation for sparse optical flow. Image Analysis and Recognition 6111:240–249.

[21] Lockwood B, Bjerke S, Kobayashi K, Guo S. (2004) Acute effects of alcohol on larval zebrafish: a genetic system for large-scale screening. Pharmacol Biochem Behav 77:647–654. 15006478 10.1016/j.pbb.2004.01.003

[22] MacPhail RC, Brooks J, Hunter DL, Padnos B, Irons TD, et al (2009) Locomotion in larval zebrafish: Influence of time of day, lighting and ethanol. Neurotoxicol 30:52–58. 10.1016/j.neuro.2008.09.011 18952124

[23] Bilotta J, Saszik S, Givin CM, Hardesty HR, Sutherland SE. (2002) Effects of embryonic exposure to ethanol on zebrafish visual function. Neurotoxicol Teratol 24:759–766. 12460658 10.1016/s0892-0362(02)00319-7

[24] Ali S, Champagne DL, Alia A, Richardson MK (2011) Large-scale analysis of acute ethanol exposure in zebrafish development: a critical time window and resilience. PLoS ONE 6:e20037. 21625530 10.1371/journal.pone.0020037 PMC3098763

[25] Kimmel CB, Ballard WW, Kimmel SR, Ullmann B, Schilling TF (1995) Stages of embryonic development of the zebrafish. Dev Dynam 203:253–310. 10.1002/aja.1002030302 8589427

[26] Edelstein A, Amodaj N, Hoover K, Vale R, Stuurman N (2010) Computer Control of Microscopes Using μManager. Curr Protoc Mol Biol 92:14.20.1–14.20.17. 10.1002/0471142727.mb1420s92 PMC3065365 20890901

[27] Lucas BD, Kanade T**.** (1981) An iterative image registration technique with an application to stereo vision. Proceedings of the 1981 DARPA Imaging Understanding Workshop: 121–130.

[28] Press WH, Flannery BP, Teukolsky SA, Vetterling WT (1989) Numerical Recipes in FORTRAN. In: The Art of Scientific Computing, Cambridge, Cambridge University Press, pp. 494–498.

[29] R Core Team (2014) R: A language and environment for statistical computing. R Foundation for Statistical Computing. Vienna, Austria. www.R-project.org.

[30] Clarke KR (1993) Non-parametric multivariate analyses of changes in community structure. Aust J Ecol 18:117–143.

[31] Minitab 16 Statistical Software, State College, PA (2010) www.minitab.com.

[32] Parng C, Roy NM, Ton C, Lin Y, McGrath P (2007) Zebrafish: A preclinical model for drug screening. J Pharmacol Toxicol Method 55:103–112. 10.1016/j.vascn.2006.04.004 16769228

[33] de Esch C, van der Linde H, Slieker R, Willemsen R, Wolterbeek A, et al (2012) Locomotor activity assay in zebrafish larvae: Influence of age, strain and ethanol. Neurotoxicol Teratol 34:425–433. 22484456 10.1016/j.ntt.2012.03.002

[34] Puttonen H, Sundvik M, Rozov S, Chen Y-C, Panula P. (2013) Acute ethanol treatments upregulated th1, th2 and hdc in larval zebrafish in stable networks. Frontiers in Neural Circuits 7:102. 23754986 10.3389/fncir.2013.00102 PMC3668275

[35] Ahmad F, Noldus LPJJ, Tegelenbosch RAJ, Richardson MK (2012) Zebrafish embryos and larvae in behavioural assays. Behavior 149:1241–1281.

[36] Noldus LP, Spink AJ, Tegelenbosch RA (2001) EthoVision: A versatile video tracking system for automation of behavioural experiments. Behav Res Methods 33:398–414. 10.3758/bf03195394 11591072

[37] Kokel D, Rennekamp AJ, Shah AH, Liebel U, Peterson RT (2012) Behavioral barcoding in the cloud: embracing data-intensive digital phenotyping neuropharmacology. Trends Biotech 30:421–425. 10.1016/j.tibtech.2012.05.001 PMC3401323 22652049

